# Advances in Optical Biosensors and Sensors Using Nanoporous Anodic Alumina

**DOI:** 10.3390/s20185068

**Published:** 2020-09-07

**Authors:** Mahmoud Amouzadeh Tabrizi, Josep Ferre-Borrull, Lluis F. Marsal

**Affiliations:** Departamento de Ingeniería Electrónica, Eléctrica y Automática, Universitat Rovira i Virgili, Avda. Països Catalans 26, 43007 Tarragona, Spain; mahmoud.amouzadeh@urv.cat (M.A.T.); josep.ferre@urv.cat (J.F.-B.)

**Keywords:** nanoporous anodic alumina, optical biosensor, immunosensor, aptasensor, peptide-based biosensor, enzyme-based biosensor

## Abstract

This review paper focuses on recent progress in optical biosensors using self-ordered nanoporous anodic alumina. We present the fabrication of self-ordered nanoporous anodic alumina, surface functionalization, and optical sensor applications. We show that self-ordered nanoporous anodic alumina has good potential for use in the fabrication of antibody-based (immunosensor), aptamer-based (aptasensor), gene-based (genosensor), peptide-based, and enzyme-based optical biosensors. The fabricated optical biosensors presented high sensitivity and selectivity. In addition, we also showed that the performance of the biosensors and the self-ordered nanoporous anodic alumina can be used for assessing biomolecules, heavy ions, and gas molecules.

## 1. Introduction

In recent years, three-dimensional nanostructures offer a new chance for researchers in the field of nanoscience to improve the performance of biodevices [1,2,3].

Self-ordered porous metal oxides (SOPMOs) are three-dimensional nanostructure platforms that are made with an electrochemical anodization method, such as tantalum [4,5,6,7,8,9], titanium [10,11,12,13], niobium [14,15,16,17], iron [18,19,20], stainless steel [21,22,23], silicon [24,25,26,27], aluminium [28,29,30,31], in acidic solutions. Among them, the self-ordered nanoporous aluminium oxide called nanoporous anodic alumina (NAA) has many advantages such as a durable platform, easy functional ability, high surface area, biocompatibility, and low-cost [32,33,34,35,36,37,38,39,40,41,42].

Several parameters should be optimized for the fabrication of NAA such as applied potential, temperature, and electrolyte. Both the inorganic acid (selenic acid [43,44], sulfuric acid [45,46], phosphoric acid [40,47]) and organic acid (oxalic acid [48,49,50,51], malonic acid [51,52], citric acid [53], etidronic acid [54,55], tartaric acid [56]) can be used as an electrolyte for the NAA fabrication.

Various applications have been reported for NAA such as biosensors [57,58,59,60,61,62,63,64,65], sensors [36,66,67,68,69,70,71], drug release [72,73,74,75,76], template-based nanowire, and nanotube fabrication [77,78,79,80]. Among the various sensors and biosensors that have been reported based on NAA, optical biosensors are the more interesting because of their remote sensing ability and properties such as being small and lightweight, having high sensitivity, and being immune to electromagnetic interference [81,82,83,84,85,86].

Up to now, several optical methods have been reported when using an NAA such as surface plasmon resonance (SPR) [87,88,89,90,91,92,93,94,95], interference localized surface plasmon resonance (ILSPR) [36,82,96,97,98,99,100,101], photoluminescence spectroscopy (PLS) [32,102,103,104,105,106,107], surface-enhanced Raman scattering (SERS) [84,108,109,110,111,112,113,114], and interferometric reflectance spectroscopy (IRS) [59,115,116,117]. Among them, IRS is a common optical method that has been applied as the basis for the NAA-based biosensors and sensors. With this method, a white light beam is illuminate toincident the SOPMO, and Fabry–Pérot (FP) interferences are obtained from the two main interfaces: (1) the interface between the incident medium and the thin film constituted by the porous structure (interface-a) and (2) the interface between the porous thin film and the substrate (usually the remaining aluminum, interface-b). In a this FP interferometer, the incident beam splits at interface-a with one part reflected back and another part transmitted into the thin film. This transmitted part of the beam travels within the thin film until it reaches interface-b, where it is reflected back. The beam travels back again to interface-a where it is split again into a transmitted portion and reflected back into the thin film portion. This process repeats itself until no energy is remaining in the beam, and a number of beams are generated in the same direction as the first reflected beam. Two consecutive reflected beams have a difference in their optical paths that depends on the angle of incidence, the effective refractive index of the thin film and its thickness.

**Figure 1:**
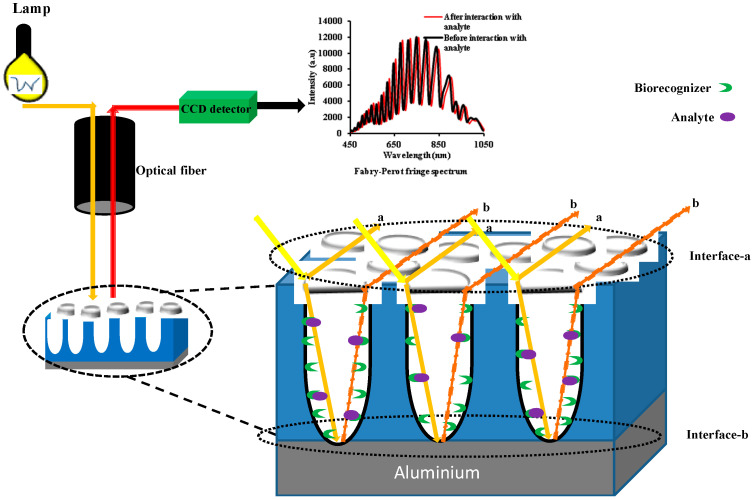
Schematic representation of the interferometric reflectance spectroscopy (IRS) detection system.

A charge-coupled device (CCD) spectrometer is used to collect and analyze these multiply reflected beams. The signal registered by the CCD depends on the optical path difference between two consecutively reflected beams. When the wavelength is such that the optical path difference is an integer number of wavelengths, the measured spectrum shows a maximum at that wavelength. On the other hand, if the optical path difference is one half of any odd integer, the specrum shows a minimum. These maxima and minima in the spextrum are known as FP fringes. Figure 1 shows the schematic of the IRS detection system.

The effective refractive index is a function of the different refractive indices composing the thin film: the oxide, the filling medium, and the attached molecules. When the pores are filled with the liquid medium and different molecules attach to the pore surfaces, the effective refractive index changes, causing a resulting shift in the FP fringes. The effective refractive index is a function of the different refractive indices composing the thin film: the oxide, the filling medium, and the attached molecules. When the pores are filled with the liquid medium and different molecules attach to the pore surfaces, such effective refractive indes changles which causes a resulting snift in the FP fringes. The amount of change depends on the concentration of the analyte in the sample.

In the next sections, first, we explain the electrochemical fabrication and functionalization of NAA. Then, we address some of the most recent papers that prove that various optical biosensors and sensors are made using NAA such as immunosensors, aptasensors, enzyme-based sensors, gas sensors, smalll biomolecule sensors, and ion sensors. We also discuss their structure, surface functionalization, method of detection, and performance parameters such as sensitivity, range of linearity, and limit of detection.

## 2. Fabrication and Functionalization of NAA

**Figure 2:**
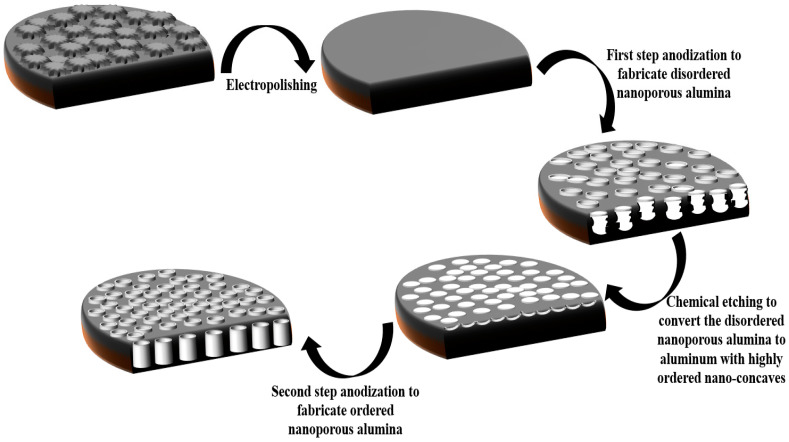
Schematic representation of the different steps to the fabrication of nanoporous anodic alumina (NAA).

The electrochemical fabrication of NAA is achieved following a two-step anodization method in electrolyte solution under stirring conditions at low temperature (5 ℃) [25,26,27,28]. To fabricate NAA, first, an aluminum sample should be electropolished in an ethanol solution containing perchloric acid (25%) to remove most of the irregularities on the Al surface. After that, the electropolished Al is immersed in an organic or inorganic electrolyte solution for the first step of the anodization process. During the first step, the Al sample corrodes and converts to disordered nanoporous anodic alumina. In order to obtain a porous thin film with good enough properties, the disordered nanoporous alumina is immersed in a phosphoric acid solution containing chromic acid to remove the porous aluminum oxide matrix leaving the aluminum surface with a highly ordered array of nano-concaves on its surface. According to reports, by anodizing Al with highly ordered nano-concaves in the same condition that is applied in the first step (second step), a highly ordered nanoporous alumina will be fabricated. The nano-concaves on AL play as the pore nucleation sites during the second step anodization. Finally, the pore widening process should be done in a phosphoric acid solution to increase the pore size. The schematic fabrication of NAA is shown in Figure 2.

**Figure 3:**
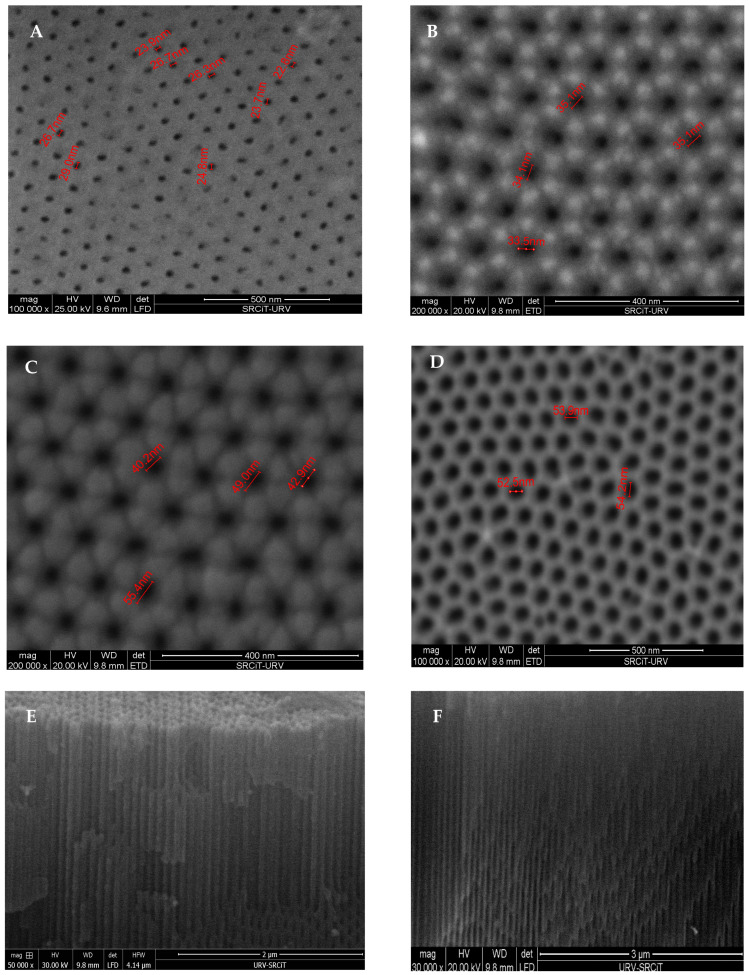
SEM images of an NAA: top views (**A**–**D**) and cross-sectional views (**E**,**F**).

The effect of the pore widening time on the pore diameters of NAA can be studied using scanning electron microscopy (SEM) techniques. Figure 3 shows SEM images of the NAA fabricated in 0.3 M oxalic acid as electrolyte solution at 40 V at various pore widening times. As can be seen, the pore diameters of the NAA increased by increasing the widening time (A–D). Also, some cross-sectional images of thee NAA are shown in Figure 3E,F. In all the cases it can be seen the uniformity of the cylindrical nanopores. By considering the top views and cross-sectional view of an NAA, it can be concluded that an NAA is a three-dimensional nanoplatform.

Since NAA does not have any functional group to interact with its biomolecules, it should first be functionalized. For this purpose, NAA was dipped into a 3.0 M hydrogen peroxide solution (H_2_O_2_) (T = 70 ℃) for 1 h to generate a hydroxide (−OH) group. The generated −OH group can interact with the silane coupling agents, such as 3-aminopropyltriethoxysilane (3-APTS) [118] 3-mercaptopropyl-tirethoxysilane (3-MPTES) [119], 3-glycidoxypropyltrimethoxysilane [120], and 3-isocyanatopropyl triethoxy (3-ISCN) [121], inducing the functional group on the pores of the NAA. Then, the silanized NAA can interact with various biomolecules such as antibodies [60], amino-terminal aptamers [122,123], enzymes [105], and peptides [124,125].

## 3. Biosensors (Biorecognizer-Based Sensors)

A biosensor consists of a bio-recognizer and a transducer that detects an analyte quantitatively and selectively. The bio-recognizers binds or reacts with an analyte specifically, causing a biological event. After that, the biological event is converted by a transducer into a diagnostic signal such as an optical signal [126].

### 3.1. Immunosensor

An immunosensor is a highly affinity-based bioanalytical device that works based on interactions between an immobilized antibody (bio-recognizers) on a transducer and an analyte in the solution [127].

Kumeria et al. showed that their microchip IRS-based immunosensor (NAA-biotinylated anti-epithelial cell adhesion molecule antibody (anti-EpCAM)) can be used for sensing circulating tumor cells (CTCs) within 5 min [128]. To fabricate the immunosensor, they first coated the surface of the NAA with a thin layer of gold (NAA-Au). Then, 11-mercaptoundecanoic acid (MUA) was self-immobilized on the surface of the NAA-Au. Afterward, the carboxylic acid group of the MUA was activated with EDC/NHS to interact with streptavidin. Finally, the biotinylated anti-EpCAM antibodies were immobilized on the surface of the NAA-Au-streptavidin based on the avidin–biotin interaction.

**Figure 4:**
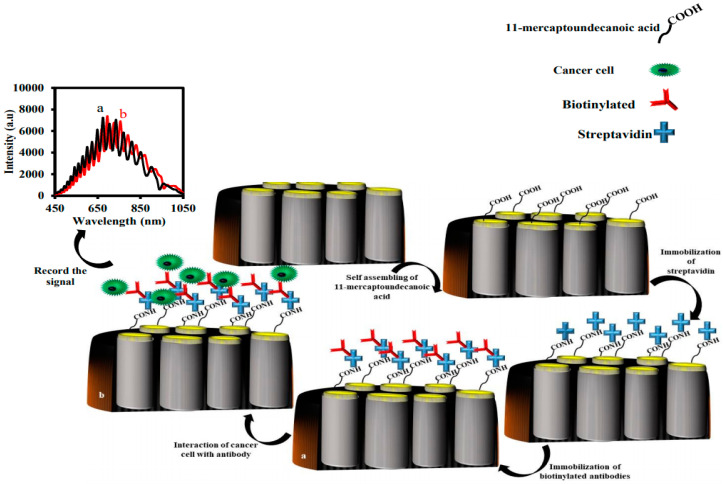
Schematic representation of the fabrication of the nanoporous anodic alumina-anti-epithelial cell adhesion molecule antibody (NAA-anti-EpCAM) for the determination of CTCs using interferometric reflectance spectroscopy (IRS).

As the CTC cell solution was injected to measure cells, they interacted with anti-EpCAM antibodies on the surface of NAA-Au. This interaction caused a redshift in the wavelength of the reflected light to the CCD detector. The amount of redshift depended on the CTC concentration in the sample. The proposed biosensor could detect the cancer cell in the concentration range of 1 × 10^3^–1 × 10^5^ cells/mL and with a detection limit of <1000 cells/mL. The schematic representation for the fabrication of the NAA-anti-EpCAM antibody and the sensing mechanism is shown in Figure 4.

In another work, Lee et al. studied the fabrication of a biosensor to detect serum amyloid A1 (SAA1) as a lung cancer-specific biomarker [98]. To detect this biomarker, the surface of the NAA was coated with Ni/Au, and then they immobilized the related antibody on it. After the injection of the SAA1 solution for measuring cells, the wavelength of the reflected light redshifted, indicating the interaction of SAA1 antigen with the immobilized antibody on the top of the NAA-Au. The results showed that the fabricated immunosensor could be used from 1 fg/mL to 1µg/mL. The LOD was found to be 100 ag/mL.

They also showed that the same strategy can be used for the determination of C-reactive protein (CRP) [129]. The linear response of the biosensor ranged from 0.1 fg/mL to 1 µg/mL, and the LOD was 100 ag/mL.

**Figure 5:**
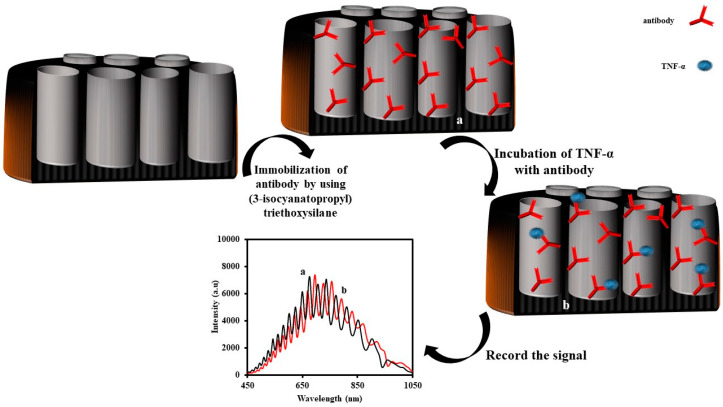
Schematic representation of the fabrication of the nanoporous anodic alumina-tumor necrosis factor alpha antibody (NAA-TNF) for the determination of tumor necrosis factor alpha using interferometric reflectance spectroscopy.

In more recent work, an IRS-based immunosensor for the determination of tumor necrosis factor alpha (α-TNF) was reported by G. Rajeev et al. [121]. The first step was the fabrication and functionalization of the NAA with 3-isocyanatopropyl triethoxy silane. The 3-isocyanatopropyl function group can interact with the primary amine of antibody, immobilizing it on the NAA. After that, they injected various concentrations of α-TNF. The results demonstrated that the wavelength of the reflected light to the CCD redshifted as the concentration of TNF increased in the sample. When α-TNF interacted with the immobilized antibody, the effective refractive index of the NAA increased, and this phenomenon caused a shift to a longer wavelength and, consequently, a shift in the effective optical thickness (EOT) according to the Fabry–Perot equation: mλ = 2 nL = EOT. In this equation, λ is the wavelength of the maximum constructive interference of order m, n is the effective refractive index of the porous film and its contents, and L is the geometric thickness of the porous thin film. To calculate the amount of change in the EOT, they applied a fast Fourier transformation to the recorded interference spectra. The results showed that the change in the EOT of NAA could be used as a signal for the measurement of α-TNF in the concentration of 100 to 1500 ng/mL. The calculated LOD was 0.13 µg/mL. The schematic representation for the fabrication of the NAA-anti-TNF and the sensing mechanism is shown in Figure 5.

### 3.2. Aptasensor

Aptasensors are a type of biosensors that use the synthesized nucleic acid sequences named aptamer as a detection element. [130]. The size of the aptamer is smaller than the antibody; therefore, in the same surface area, the amount of the immobilized detection element in the aptasensor is higher than the immunosensor and, subsequently, the linear response range of aptasensors, in the most of the times are wider than immunosensor [131]. In addition, instead of immunosensors that can be used for sensing the limited number of targets, aptasensors can be used for a large number of targets from cancer cells to ions. In this section, some of the more interesting aptasensors that have been made using NAA are collected.

Silicon nanoporous substrates are common materials that have been used for molecular gate-based aptasensing purposes [132]. In these kinds of sensing strategies, silicon nanoporous particles should be first loaded with dye molecules such as rhodamine B. Then, the entrance of the pores of the silicon nanoporous particles are functionalized with a silane coupling agent, such as (3-isocyanatopropyl)triethoxysilane, to attach the short single-stranded DNA sequences (normally from 4 to 12 oligonucleotide sequences). The first oligonucleotide sequences and last oligonucleotide sequences of the aptamer probe can interact complementary to the single-stranded DNA, immobilizing the aptamer probe, therefore aptamer. To explain it more clearly, in this sensing strategy, nanoporous particles and an aptamer play like a pot and a lid, respectively, and inside the pot (nanoporous particles) is filled with dye. If any analyte can interact with the aptamer probe selectively, so, the dye will release on nanoporous particles and then the color of the solution will change. If the length of the aptamer probe is smaller than the pore diameter of the nanoporous substrate, the dye molecule will not remain inside the porous and will release into solution. Hence, the length of the aptamer probe must be long enough to cover the pore diameter of the nanoporous substrate. Since the length of the aptamer is small (up to 10 nm), meso-sized porous substrates can be used for molecular gate-based sensing. Among the different nanoporous materials, NAA is a good candidate.

Nanoporous anodic alumina (NAA) fabricated in a sulphuric acid electrolyte can have a porous structure with an average pore size of 8 nm, making it a good candidate for molecular gate-based sensing. In this context Martínez et al. used NAA and molecular gates for the detection of cocaine using rhodamine B and the photoluminescence spectroscopy (PLS) method as a molecular probe and a sensing device, respectively [129]. The sequence of short single-stranded DNA (i.e., 5′-AAAAAACCCCCC-3′) was immobilized in the entrance of the nanopore using (3-isocyanatopropyl) triethoxysilane, and the sequence of the aptamer probe of cocaine was TTTTGG GGGGGG GAG ACA AGG AAA ATC CTT CAA TGA AGT GGG TCT CCA GGGGGGTTTT-3.

**Figure 6:**
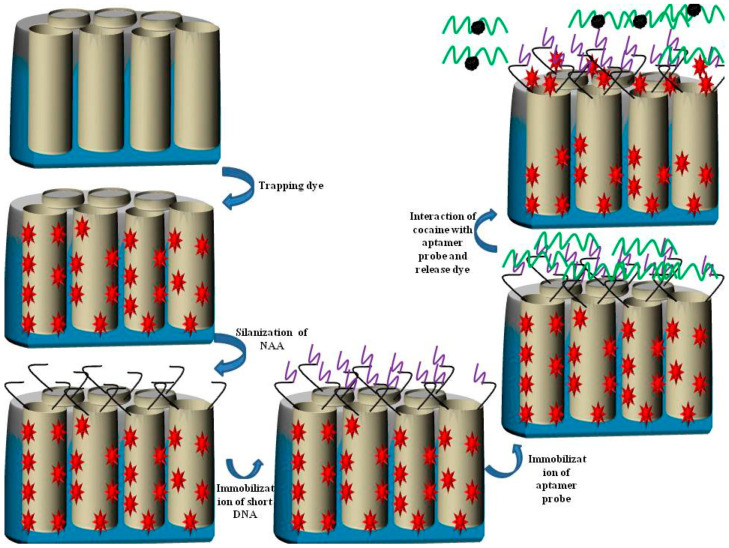
Schematic representation of the fabrication of the NAA-short chain aptamer/aptamer_probe_ for the determination of cocaine using photoluminescence spectroscopy (PLS).

As demonstrated, the first oligonucleotide sequences and last oligonucleotide sequences of an aptamer probe interacted complementarily to single-stranded DNA, immobilizing the aptamer probe in the entrance of the pores of NAA. As the aptamer probe interacted with the cocaine, it removed from the NAA, and then rhodamine B was released into the solution. The amount of the released rhodamine B increased with the increase in the concentration of cocaine. The signal of the released rhodamine B was detected by the fluorescence spectrometer. The results showed that the proposed method could detect cocaine concentrations of 0.5 µM to 1 mM. The LOD was 0.5 µM and the sensor had a higher affinity to cocaine than morphine and heroin. The schematic representation for the fabrication of the aptasensor is shown in Figure 6.

In another work, an interferometric reflectance spectroscopy (IRS)-based aptasensor for the determination of thrombin (TB) [123] has been reported by Pol et al. For that purpose, NAA was silinzed with 3-APTS. The amino-functionalized NAA then interacted with the sulfo-NHS-biotin. According to previous reports, the amino-functional group can interact with streptavidin. To immobilize the aptamer probe, they used a biotinylated aptamer probe to interact with immobilized streptavidin. The results showed that the wavelength of the reflected light from the proposed aptasensor to the CCD shifted to a high wavelength (redshift) in the presence of TB, indicating the change in the effective optical index of NAA after the incubation of the TB with aptamer. The proposed sensor can be used for sensing TB in a concentration range of 0.54–2.70 µM. The experimental detection limit was 7.2 nM.

Recently, a novel strategy for the quantitive measurement of a target using the IRS technique was reported by Tabrizi et al. [130]. The sensitivity of the aptasensor would be higher if the intensity of the reflected light could be changed during the sensing process instead of the wavelength of the reflected light.

For this purpose, an IRS-based biosensor was fabricated using NAA and functionalized with 3-APTS. A guanine-rich aptamer was used as a probe and methylene blue as a photo-probe. Amino-terminal guanine-rich aptamers were immobilized on the NAA-NH_2_ using glutaraldehyde (Glu) as the cross-linking agent. Following this, methylene blue (MB) was intercalated in the aptamer and generated the first MB/G-quadruplex complex-based IRS biosensor. A guanine-rich aptamer can fold into a G-quadruplex structure in the presence of K^+^ ion. The G-quadruplex has a very high binding ability towards MB. Besides, an MB that has a high absorption coefficient (ε = 95000 L × mol^−1^ × cm^−1^) can absorb light in the range of 550–725 nm. Therefore, the intensity of the reflected light from the MB/G-quadruplex complex-based IRS biosensor would be low compared with a common aptamer-based IRS biosensor.

Amyloid β (Aβ) oligomers were selected as a model analyte. To measure the Aβ oligomers’ concentration, the first step was to record the signal of the MB/G-quadruplex complex-based IRS biosensor in the absence of analyte. After the injection of the Aβ oligomers solution, the intensity of the reflected light to the CCD detector increased because of the release of MB on the MB/quadruplex complex. The decrease in light intensity led to a decrease in the peak area in the range of 450–1050 nm.

**Figure 7:**
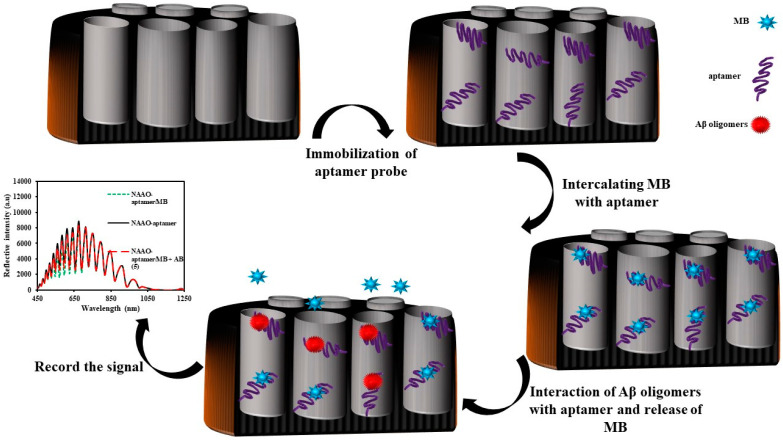
Schematic representation of the fabrication of the NAA-aptamer/MB for the determination of Aβ oligomers using IRS.

In this study, the slope of the calibration curve obtained with this method was compared with the slope of the calibration curve obtained with a common method based on the change in the EOT. The results indicated that not only was the slope of the plot obtained with the new method higher, but also the linear range of plot was wider. Consequently, the analytical performance of the sensor would be better if the ∆peak area, as a signal, was used to detect the analyte. The sensor showed a determination of Aβ oligomers in the range of 0.5 to 50.0 µg × mL^−1^, and the LOD was 0.02 µg/mL. The proposed sensor has two advantages: first, the sensitivity of the sensor-based IRS was improved; and second, the data processing step for obtaining the signal was simpler. The schematic representation for the fabrication of the NAA-Aptamer/MB and the sensing mechanism is shown in Figure 7.

**Figure 8:**
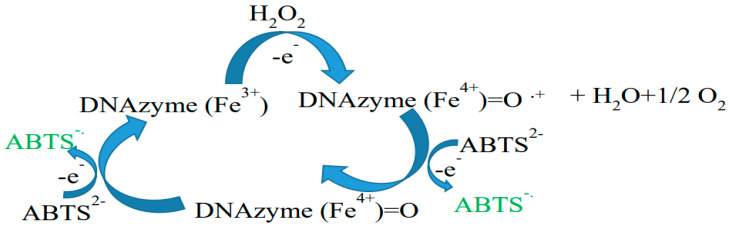
The mechanism of oxidation of ABTS^2−^ by DNAzyme.

DNAzyme is an artificial enzyme that is made of hemin and aptamer [133]. Like peroxidase enzyme, DNAzyme that has hemin cofactor can oxidase ABTS^2−^ ion to ABTS^−^ (Figure 8).

**Figure 9:**
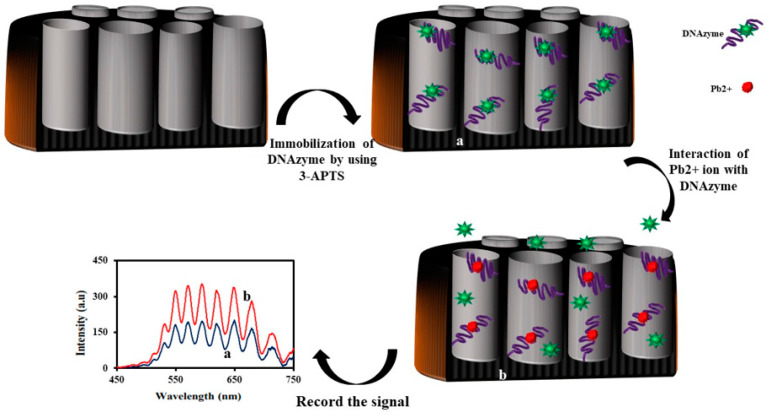
Schematic illustration of the NAA-DNAzyme sensor for the determination of Pb^2+^ ions using IRS.

Recently, a DNAzyme-based IRS biosensor to detect Pb^2+^ ion concentration in the nanomolar range was reported [134]. In this work, NAA was fabricated using a two-step anodization process, silanizng the NAA pores with 3-APTS, and followed by immobilizing the glutaraldehyde cross-linker. Therefore, they put the functionalized nanosubstrate into an amino-terminal G-rich aptamer solution containing hemin and potassium ions. According to the previous reports [96,135], the G-rich aptamer has been folded into a G-quadruplex structure named the K^+^-stabilized G-quadruplex. The K^+^-stabilized G-quadruplex shows a high binding ability towards the hemin cofactor. Therefore, in the presence of the G-rich aptamer, K^+^ ion, and hemin, an artificial peroxidase enzyme-based biosensor was fabricated. Peroxidase enzymes can oxidize 2,2′-azino-bis(3-ethylbenzothiazoline-6-sulphonic acid) (ABTS) solution in the presence of hydrogen peroxide, generating a green colored ABTS^−^ anion radical solution which can absorb light in the 45–850 nm wavelength range. The idea is to record the artificial enzyme-based biosensor K^+^-stabilized hemin-G-quadruplex DNAzyme using the IRS method. The results showed that the intensity of the reflected light from the biosensor to the CCD detector decreased when hydrogen peroxide was injected into the solution. It proved that the DNAzyme-based IRS biosensor worked properly. The generated green colored ABTS^−^ anion radical solution absorbed the white light and, therefore, the intensity of the reflected light from the biosensor to the CCD decreased. However, in the presence of Pb^2+^ ions, the catalytic property of the proposed biosensor decreased. As they added Pb^2+^ ions into the solution, the intensity of the reflected light to the CCD detector increased. In the presence of Pb^2+^ ion, the K^+^-stabilized hemin-G-quadruplex DNAzyme re-designed to the Pb^2+^-stabilized G-quadruplex. During this process hemin, the cofactor left the structure and washed away. Therefore, compared to the K^+^-stabilized hemin-G-quadruplex DNAzyme, the Pb^2+^-stabilized G-quadruplex did not show any peroxidase-like activity to oxide ABTS. Hence, the signal intensity increased. The results demonstrated that the fabricated biosensor can be used for the determination of Pb^2+^ ion in the nanomolar to the micromolar range (50–3200 nM). The low detection limit was calculated at 12 nM. The sensitivity of the proposed IRS-based sensor was higher than previous IRS-based sensors that have been used for heavy metal sensing [136,137,138,139,140,141]. It was also investigated the selectivity of the Pb^2+^ ion sensor towards interfering ions, such as Zn^2+^, Cu^2+^, Ni^2+^, Bi^3+^, Sn^2+^, Co^2+^, and Hg^2+^, in the presence of SCN^−^ ions as a masking agent and also as a biosensor for the determination of Pb^2+^ ions in seawater. The proposed IRS biosensor presented a good selectivity, and the obtained recoveries were in the range of 93.8–110.2%. To sum up, the prepared biosensor exhibited high sensitivity, good selectivity, and applicability to Pb^2+^ ion in real samples. The schematic representation for the fabrication of the NAA-DNAzyme and the sensing mechanism is shown in Figure 9.

### 3.3. Genosensor

Like an aptasenor, a genosensor uses synthesized nucleic acid sequences as a biological recognition element. But they are applied for sensing the RNA or DNA of targets such as bacteria [142] and viruses [143].

Candida albicans is one of the most common infectious fungi that can live inside the human body. Ribes et al. presented a molecular gate-based sensing method for the determination of Candida albicans-specific DNA fragments [144]. The proposed genosensor could detect Candida albicans within a20 min in the range of 7−2 × 10^2^ CFU/mL. The LOD was 8 CFU/mL.

**Figure 10:**
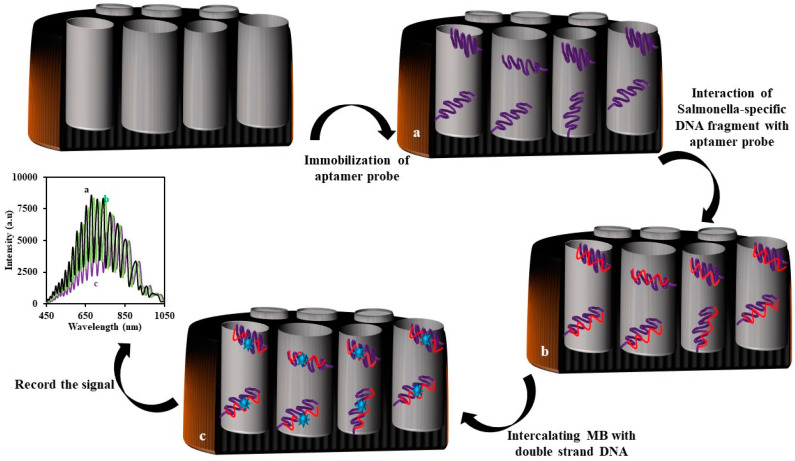
Schematic representation of the genosensor for the determination of a Salmonella-specific DNA fragment using IRS.

In another recent paper, Tabrizi et al. designed a novel IRS-based genosensor for sensing Salmonella-specific DNA fragments as a model for DNA targets [145]. To fabricate the genosensor, first they immobilized the amino-terminal DNA probe inside the pores of the NAA using Glu as a crosslinker. Next, a known concentration of Salmonella-specific DNA fragment was added into the flow cell. After washing with deionized water, methylene blue solution was added into the flow cell. Finally, the fabricated aptasensor (NAA-Aptamer-MB) was washed several times to remove all the loosely attached MB. According to previous reports [146,147], methylene blue can intercalate between cytosine and a guanine bond, immobilizing on the genosensor. The intercalated methylene blue then absorbs the illuminating white light to the genosensor. Therefore, the intensity of the reflected light from genosensor to the CCD detector decreased. The decrease in the intensity of the reflected biosensor that was considered a signal had a logarithmic relationship within the range of 0.25–50.0 nM. The limit of detection was found to be 0.01 nM. A schematic representation for the fabrication of the genosensor is shown in Figure 10.

### 3.4. *Peptide*-Based Biosensor

A peptide-based biosensor is a type of biosensor where the synthesized peptide fragments or natural proteins, as biorecognition elements, are used as bio-recognizers to interact with the target. These kinds of sensors can be used for various targets [148].

Nemati et al. reported an IRS-based biosensor using a gelatin-modified NAA for the determination of trypsin enzyme as a protease enzyme [124]. Gelatin is a polypeptide that trypsin can cleave to in small fragments at the carboxyl-terminal side of lysine and arginine residues. To fabricate the biosensor, 3-APTS and glutaraldehyde were used to immobilize gelatine on the pore of the NAA. The measurements showed that the length of the reflected light shifted to a high wavelength, as gelatin immobilized on the pore of the NAA. However, as enzyme trypsin was injected into the measuring cell, the wavelength of the reflected light blueshifted, indicating the cleavage of the gelatine and release the small peptide fragments into solution. The results demonstrated that the fabricated sensor can be used for the determination of trypsin enzymes in a concentration range of 0.0125–1 mg/mL and with 0.025 mg/mL. This result indicates that the sensitivity of the biosensor is still low for the determination of trypsin in real samples and further developments are needed.

**Figure 11:**
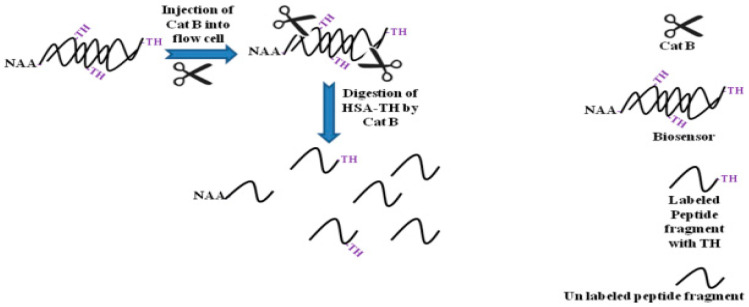
Schematic representation for the sensing mechanisms of cathepsin B (Cat B).

Another interesting example is the development of an IRS biosensor for sensing a protease enzyme named cathepsin B (Cat B) [149]. For this purpose, the NAA surface was modified with 3-APTS and human serum albumin labeled with thionin (HSA-TH). The HSA-TH played the main role in the proposed biosensor. Because the absorption coefficient of TH is so high (5.85 × 10^4^ dm^3^·mol^−1^·cm^−1^) and can absorb light in the range 510–640 nm. Therefore, the immobilized TH adsorbed the light and the intensity of the reflected light from biosensor (NAA-HSA-TH) to the CCD detector was low in the absence of Cat B. In the presence of Cat B, peptides like HSA-TH cleaved to small peptide fragments. As the TH-peptide fragment washed away from the measuring cell, the intensity of the reflected light to the CCD detector increased. A schematic diagram of the sensing process is shown in Figure 11.

**Figure 12:**
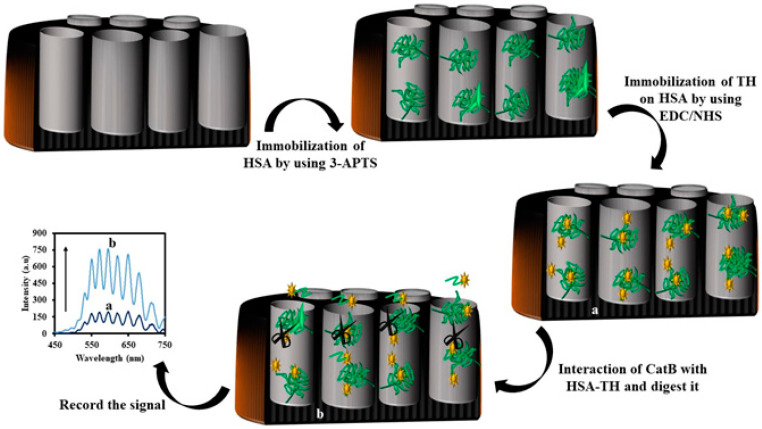
Schematic design of the nanoporous anodic alumina-human serum albumin-thionine (NAA-HSA-TH) for the determination of cathepsin B (Cat B) using interferometric reflectance spectroscopy (IRS).

The proposed sensor can be used for the measurement of Cat B in the range of 0.5–64.0 nM. The limit of the detection of the biosensor was 0.08 nM. The good selectivity towards Cat B was also demonstrated by analyzing the interfering effect of some biomolecules such as proteases trypsin enzyme, urokinase enzyme, urea, glucose, and dopamine. The schematic representation for the fabrication of the NAA-HSA-TH and the sensing mechanism is shown in Figure 12.

### 3.5. Enzyme-Based Biosensor

An enzyme-based biosensor was considered the first biosensor that was fabricated. In these types of biosensors, an enzyme is immobilized on the surface of the transducer. An enzyme is a bio-recognizer that can catalyze a target selectively [150].

A highly sensitive enzyme-based biosensor was proposed for IRS sensing of cytochrome c (Cyt C) by immobilizing trypsin (Tryp) enzyme on NAA [151]. To fabricate the enzyme-based biosensor, it was silanized NAA with 3-APTS (NAA-NH_2_). Then, the NAA-NH_2_ was immersed in a glutaraldehyde solution. After that, the Tryp enzyme solution was dropped on it to immobilize the Tryp on the pores of the NAA. According to previous reports, the Tryp enzyme can cleave Cyt C to the short peptide [152] fragments, and some of those fragments have a hemin cofactor. The structure of hemin–peptide fragments which are generated by Tryp is as follows:

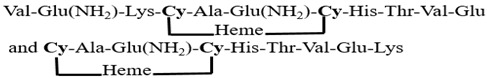


**Figure 13:**
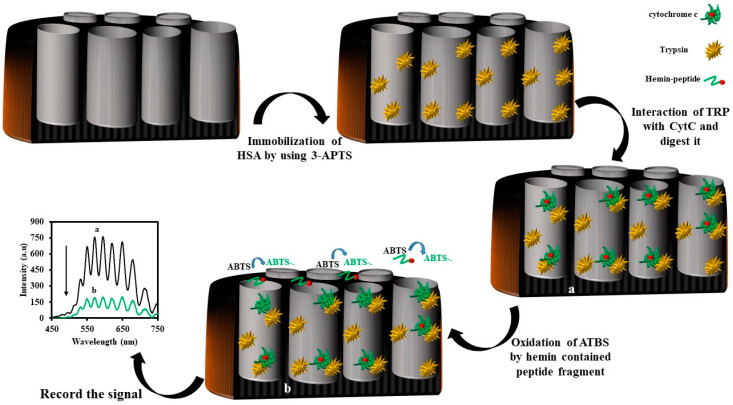
Schematic design of the nanoporous anodic alumina-trypsin (NAA-Try) biosensor for the determination of cytochrome c (Cyt C) using IRS.

In the presence of hydrogen peroxide, the heme–peptide fragments oxidized 2,2′-azino-bis(3-ethylbenzothiazoline-6-sulphonic acid) (ABTS) to a green colored ABTS^·−^ anion radicals [153]. The generated green colored ABTS^·−^ anion radical solution adsorbed the light. Hence, the intensity of the reflected light from NAA to the CCD decreased. The results revealed that the biosensor can be used for sensing Cyt C in the range of 1–100 nM, and the limit of detection is 0.5 nM. In addition, the analytical performance of the biosensor in real samples exhibited high selectivity, sensitivity, and good stability. The recovery of the analysis was 97.6%. A schematic representation of the design of the NAA-Try biosensor and the sensing mechanism is shown in Figure 13.

Another example of an enzyme- and IRS-based sensor is the urea biosensor [154]. The authors showed that the change in the intensity of the reflected light can be used for the determination of small molecules such as urea. In particular, fluorescein 5(6)-isothiocyanate (FLITC) attached to the urease enzyme could be used for the measurement of urea in the real sample. As per previous reports, in a high pH of the solution, FLTC can adsorb a greater intensity of light and, therefore, a limited fluorescence light of it would be high in compensation in high pH solution. Therefore, FLITC is considered a pH-sensitive photo probe.

As a consequence, a urease enzyme was immobilized on NAA and then attached to FLITC on an enzyme. Since the isothiocyanate functional group can interact with –NH_2_, –SH, and OH groups, therefore FLITC can attach to enzymes that have a massive number of these groups. During the catalytic process of urea by urease enzymes, ammonia is generated [155]. As ammonia is considered a base, the pH of the solution will increase too. Consequently, the absorption light property of the attached FLITC on a biosensor (NAA–Urease–FLTC) will change. Because of that, the intensity of the reflected light from the NAA–urease–FLTC sensor to the CCD detector decreases. The proposed biosensor can be used for sensing urea in the range of 0.12 to 3.0 mM with a limit of detection of 0.06 mM.

**Figure 14:**
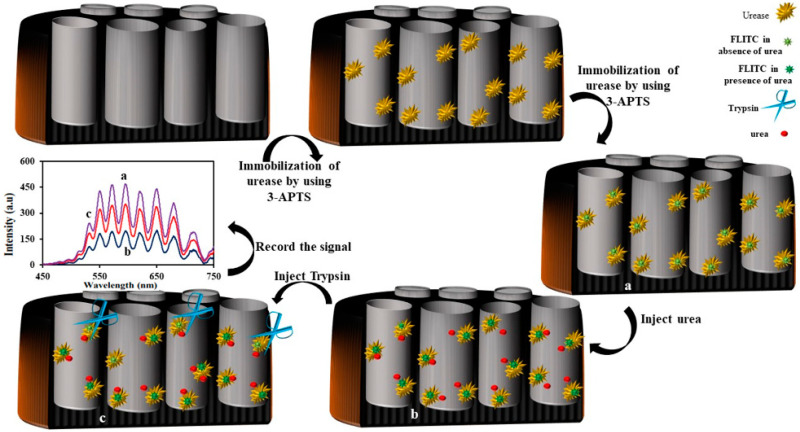
Schematic illustration showing the fabrication of the nanoanodic alumina-Urease- fluorescein 5(6)-isothiocyanate (NAA-Urease-FLITC) biosensor for the determination of trypsin using interferometric reflectance spectroscopy (IRS).

The analysis of the results also demonstrated that the catalytic activity of the urease enzyme in the presence of trypsin as the protease enzyme would change [156]. In the presence of trypsin, the activity of urease decreased and the amount of the catalyzed urea to hydroxide ion and carbon dioxide decreased. Since the amount of the generated hydroxide ion decreased, the pH solution of the solution did not increase. In this condition, the FLITC did not absorb more light and, consequently, the CCD detector detected more light. The proposed biosensor exhibited a good response to a concentration of trypsin in the range of 1.0–6.0 μg/mL. The limit of detection (LOD) for trypsin was 0.23 μg/mL, respectively. The Michaelis-Menten constant (K_m_) was calculated to be 0.078 mM for urea. The half-maximal inhibitory concentration of trypsin (IC_50_) for the proposed biosensor was 6.2 µg/mL. The proposed biosensor exhibited good selectivity, linear range responsibility, and stability. The schematic representation for the fabrication of the NAA–Urease–FLITC and the sensing mechanism is shown in Figure 14.

## 4. Sensors

### 4.1. Gas Sensor

Kumeria et al. reported the use of NAA in an IRS-based gas sensor for the determination of hydrogen sulfide (H_2_S) and hydrogen (H_2_) gas [115]. The authors proved that if the surface of the NAA was coated with gold and platinum using a sputtering method, the fabricated sensor could be used for H_2_S and H_2_ sensing respectively. The optimum pore size and thickness of the porous layer were 30 nm and 4 µm, respectively. The fabricated sensor could detect the concentration of gas in the air up to 2%.

### 4.2. Non-Biorecognizer-Based Sensor

**Figure 15:**
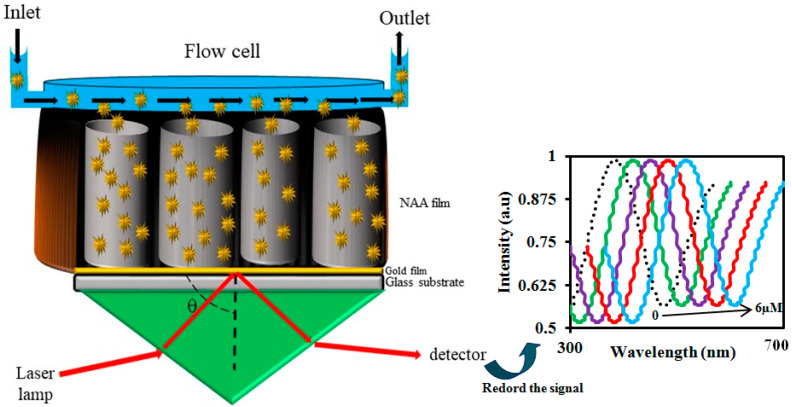
Schematic representation of the nanoporous optical waveguide (NPWG)-based sensing of bovine serum albumin (BSA) using nanoporous anodic alumina (NAA).

Teramae et al. designed and fabricated NAA to detect bovine serum albumin (BSA) by using nanoporous optical waveguide (NPWG) spectroscopy [86]. In the NPWG, like the conventional surface plasmon resonance (SPR), the changes in reflection spectra of the SOPMO film are measured in the Kretschmann configuration. The authors engineered two kinds of NAA substrate. The thickness and pore size of the substrate A was 220 nm and 39 nm, respectively, and its porosity was 34%. The thickness and pore size of the substrate B was 670 nm and 33 nm, respectively, and its porosity was 45%. According to the results, the sensor response of the NAA film B that had more porosity was about seven times larger than NAA film A due to the fact of its large adsorption capacity. The results confirmed that the wavelength of the recorded signal redshifted in the presence of BSA. The fabricated sensor could detect BSA concentration from 60 nM to 60 µM and the limit of detection is was 5.7 pg/mm^2^. The schematic representation for the NPWG sensing of BSA is shown in Figure 15.

In another article, Chen et al. reported an IRS-based sensor for the determination of vitamin C [157]. To fabricate the sensor, first, the NAA was functionalized with 3-APTS, and then glutaraldehyde was immobilized on the NAA pores. The sensing was obtained when the vitamin C interacted with glutaraldehyde, changing the EOT of the NAA. The fabricated sensor showed a detected concentration of vitamin C ranging from 0.125 µM to 0.5 µM and a limit of detection of 20 nM.

### 4.3. Ion Sensor

Kumeria et al. presented a nanoporous interferometric sensor for label-free detection of gold (III) [138]. Nanoporous anodic alumina (NAA) was used as a nanoporous material and was functionalized with 3-mercaptopropyl-triethoxysilane. The selectivity of gold (III) ion was evaluated in the presence of some heavy metals such as Fe^3+^, Mg^2+^, Co^2+^, Cu^2+^, Ni^2+^, Ag^+^, and Pb^2+^ ions. The fabricated sensor detected the concentration of Au^3+^ (III) ion in the range of 0.1–80 μM with a lower detection limit of 0.1 μM. The interaction of the thiol (–SH) group of 3-MPTES with Au^3+^ (III) ion played a key role in this sensor. According to their report, the experimental data were fitted with Langmuir isotherm model binding better than Freundlich isotherm, suggesting absorption of Au^3+^ ions on NAA-3-MPTES.

**Figure 16:**
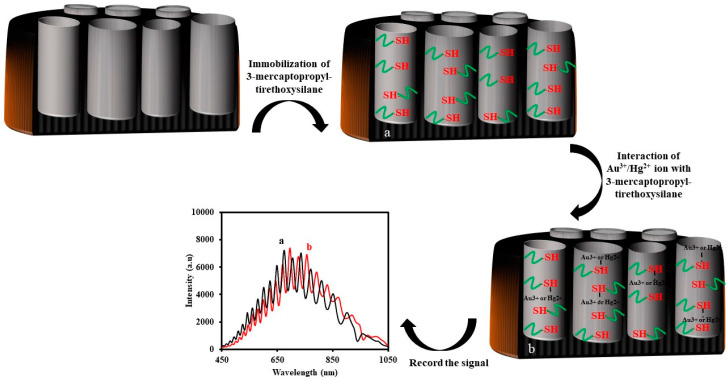
Schematic representation for the fabrication of the interferometric reflectance spectroscopy IRS based sensor for mercury (II) Hg^2+^ or gold ion (III) Au^3+^ ion sensing.

Furthermore, they also reported that the immobilized 3-mercaptopropyl-tirethoxysilane on the NAA with rugate structure could be used for the determination of Hg^2+^ ion in the range of 1–100 µM [136]. The schematic representation for the fabrication of the NAA-3-MPTES and the sensing mechanism of Au^3+^ or Hg^2+^ ions is shown in Figure 16.

## 5. Conclusions

This review article highlights recent advances in optical biosensors based on nanoporous anodic alumina. Detailed information was presented about the fabrication, structure, surface modification, and biosensing properties of nanoporous anodic alumina. We also provided fundamental aspects of optical techniques, such as interferometric reflectance spectroscopy, surface plasmon resonance, and photoluminescence spectroscopy, in combination with NAA platforms and discussed some relevant examples of optical NAA-based immunosensors, aptasensors, peptide-based biosensors, and genesensors.

In general, the optical sensors and biosensors based on NAA and new sensing strategies, such as plasmonic and interferometric spectroscopy, offer high sensitivity and low detection limits and present interesting features such as being remote, cheap, and integratable into lab-on-chip systems. The NAA-based sensors and biosensors have a high potential for the determination of a wide size range of analyte from big bio-components, such as cancer cells, to small ones such as glucose.

Advances and developments in NAA optical structures and the versatility to modify the surface of NAA for new functionalities with specific selectivity will provide a new generation of NAA-based sensing systems with better performance and their use as a point-of-care testing devices. Finally, Table 1 summarizes an updated list of reported NAA-based optical biosensors and sensors

## Figures and Tables

**Table 1:** The analytical performance of the optical biosensors and sensors by using NAA.

Sensor	Type of Recognizers	Analyte	Method	Linear Range	LOD	Ref.
Nanoporous anodic alumina - Human serum albumin-Thionine	Labeled Peptide (Human serum albumin-Thionine)	Cathepsin B	Interferometric reflectance spectroscopy	0.5–64.0 nM	0.08 nM	[137]
Nanoporous anodic alumina -Urease- Fluorescein 5(6)-isothiocyanate	Labeled Enzyme (Urease- Fluorescein 5(6)-isothiocyanate)	Trypsin	Interferometric reflectance spectroscopy	0.25–20 μg/mL	0.06 μg/mL	[156]
Nanoporous anodic alumina-Gelatin	Peptide (Gelatin)	Trypsin	Interferometric reflectance spectroscopy	1–7 mg/mL	1 mg/mL	[124]
Nanoporous anodic alumina-Trypsin	Enzyme (Trypsin)	Cytochrome c	Interferometric reflectance spectroscopy	1–100 nM	0.5 nM	[151]
Nanoporous anodic alumina -ssDNAsal	Short chains of nucleotides (ssDNAsal)	Salmonella-specific DNA fragment	Interferometric reflectance spectroscopy	0.25–50.0 nM	0.01 nM	[145]
Nanoporous anodic alumina -Aptamer_TB_	Short chains of nucleotides (AptamerTB)	Thrombin	Interferometric reflectance spectroscopy	0.54–2.70 nM	7.2 nM	[123]
Nanoporous anodic alumina -Aptamer_Aβ_	Short chains of nucleotides (AptamerAβ)	Amyloid β oligomers	Interferometric reflectance spectroscopy	0.5–50.0 μg/mL	0.02 μg/mL	[122]
Nanoporous anodic alumina -Anti- Tumor necrosis factor alpha	Antibody (Anti- Tumor necrosis factor alpha)	Tumour necrosis factor-alpha	Interferometric reflectance spectroscopy	100–1500 ng/mL	100 ng/mL	[121]
Nanoporous anodic alumina- Anti- Epithelial cell adhesion molecule antibody	Antibody (Anti- Epithelial cell adhesion molecule antibody)	Circulating tumor cells	Interferometric reflectance spectroscopy	103–105	>1000	[128]
Nanoporous anodic alumina-Anti- human immunoglobulin G	Antibody (Anti- human immunoglobulin G)	Human immunoglobulin G	Interferometric reflectance spectroscopy	10–100 μg/mL	1 μg/mL	[158]
Nanoporous anodic alumina -Streptavidin	Peptide (Streptavidin)	Biotinylated thrombin	Interferometric reflectance spectroscopy	10–100 μg/mL	10 μg/mL	[159]
Nanoporous anodic alumina gradient-index	-	Glucose	Interferometric reflectance spectroscopy	0.025–1 M	0.025 M	[160]
Nanoporous anodic alumina -3-Aminopropyltriethoxysilane -Glutaraldehyde	Small molecule (Glutaraldehyde)	Vitamin C	Interferometric reflectance spectroscopy	0.125–0.5 µM	20 nM	[157]
Nanoporous anodic alumina	-	Glucose	Interferometric reflectance spectroscopy	0.0125–1 M	0.0125	[161]
Nanoporous anodic alumina	-	Glucose	Interferometric reflectance spectroscopy	0.01–1.2 M	100 mM	[162]
			Photoluminescence spectroscopy	0.01–1.2 M	10 mM	
Nanoporous anodic alumina	-	L-cysteine	Interferometric reflectance spectroscopy	0.005–0.1 M	5 mM	[162]
			Photoluminescence spectroscopy	0.005–0.1 M	5 mM	
Nanoporous anodic alumina	-	Glucose	Photoluminescence spectroscopy	0.01–1.1 mM	0.01 mM	[163]
Nanoporous anodic alumina -3-Mercaptopropyl-tirethoxysilane	Small molecule (3-Mercaptopropyl-tirethoxysilane)	Mercury(II) ion	Interferometric reflectance spectroscopy	1–100 μM	1 μM	[136]
Nanoporous anodic alumina - Polyethylenimine- Glutaraldehyde-Polyethylenimine	Polymer (Polyethylenimine - Glutaraldehyde - Polyethylenimine)	Copper (II) ion	Interferometric reflectance spectroscopy	1–100 mg/L	0.007 mg/L (7 ppb)	[137]
Nanoporous anodic alumina -3-Mercaptopropyl-tirethoxysilane	Small molecule 3-Mercaptopropyl-tirethoxysilane	Glod (III) ion	Interferometric reflectance spectroscopy	0.1–80 µM	0.1 µM	[138]
Nanoporous anodic alumina -DNAzyme	Short chains of nucleotides and heme group (DNAzyme)	Lead ion (II)	Interferometric reflectance spectroscopy	50–3200 nM	12 nM	[134]
Nanoporous anodic alumina-Bovine serum albumin-5- Fluorouracil	Labeled Protein (Bovine serum albumin-5- Fluorouracil)	Fluorouracil antibody	Interference localized surface plasmon resonance	10–10^4^ ng/mL	10 ng/mL	[96]
Nanoporous anodic alumina-Human serum albumin	Protein (Human serum albumin)	Quercetin	Interferometric reflectance spectroscopy	0.25–0.5 mg/mL	0.14 mg/mL	[164]
Nanoporous anodic alumina- poly(acrylic acid) [poly(acrylic acid)/protonated poly(allylamine)]3	poly(acrylic acid)/protonated poly(allylamine)	Cy5-labeled human immunoglobulin G	Photoluminescence spectroscopy	0.02–1 ng/mL	0.02 ng/mL	[165]
Nanoporous anodic alumina	-	Bovine serum albumin	Nanoporous optical waveguide	60 nM–6 µM	5.7 pg/mm^2^	[86]
Nanoporous anodic alumina-short aptamer/Rhodamine B sequence/Aptamer_Cocaine probe_	Short chains of nucleotides (aptamerCocaine probe)	Cocaine	photoluminescence spectroscopy	0.5–10 µM	0.5 µM	[166]
Nanoporous anodic alumina-short aptamer/Rhodamine B sequence/Aptamer probe	Short chains of nucleotides	Mycoplasma species genome	Photoluminescence spectroscopy	20–80 copies/mL	20 copies/mL	[102]
Nanoporous anodic alumina-short aptamer/Rhodamine B sequence/Aptamer_Candida albicans species probe_	Short chains of nucleotides (AptamerCandida albicans speciesprobe)	Candida albicans species genome	Photoluminescence spectroscopy	7−2 × 10^2^ CFU/mL	8 CFU/mL	[144]
Nanoporous anodic alumina-short aptamer/Rhodamine B sequence/Aptamer _Staphylococcus aureus species genome probe_	Short chains of nucleotides (Aptamer Staphylococcus aureus species)	Staphylococcus aureus species genome	Photoluminescence spectroscopy	2–100 CFU/mL	2 CFU/mL	[167]
Nanoporous anodic alumina -biotin-Stripavidin/Aptamer probe	Short chains of adenines	Timine rich oligumer	Optical waveguide	50 pM–1 nM	20 pM	[91]

## Acronyms

SOPMO	Self-ordered porous metal oxides
Al	Aluminum
NAA	Nanoporous anodic alumina
3-APTS	3-Aminopropyltriethoxysilane
3-MPTES	3-Mercaptopropyl-tirethoxysilane
3-ISCN	3-Isocyanatopropyl triethoxy
PLS	photoluminescence spectroscopy
IRS	Interferometric reflectance spectroscopy
SERS	Surface-enhanced Raman scattering
CCD	Charge-coupled device
α-TNF	Tumor necrosis factor alpha
anti-EpCAM	Anti- Epithelial cell adhesion molecule antibody
CTC	Circulating tumor cells
Glu	Glutaraldehyde
MB	Methylene blue
TB	Thrombin
EOT	Effective optical thickness
Aβ	Amyloid β
ABTS	2,2′-azino-bis(3-ethylbenzothiazoline-6-sulphonic acid)
CFU	Colony-forming unit
Cat B	Cathepsin B
Cyt C	Cytochrome c
FLITC	Fluorescein 5(6)-isothiocyanate
K_m_	Michaelis-Menten constant
H_2_S	Hydrogen sulphide
H_2_	Hydrogen
PEI	Polyethylenimine
Rh B	Rhodamine B
OWG	Optical waveguide

## References

[1] Wang J., Karnaushenko D., Medina-Sánchez M., Yin Y., Ma L., Schmidt O.G. (2019). Three-Dimensional Microtubular Devices for Lab-on-a-Chip Sensing Applications. ACS Sens..

[2] Santos A., Kumeria T., Losic D. (2013). Nanoporous anodic aluminum oxide for chemical sensing and biosensors. TRAC-Trend Anal. Chem..

[3] Aw M.S., Bariana M., Losic D., Losic D., Santos A. (2015). Nanoporous Anodic Alumina for Drug Delivery and Biomedical Applications. Nanoporous Alumina: Fabrication, Structure, Properties and Applications.

[4] Sieber I., Hildebrand H., Friedrich A., Schmuki P. (2006). Initiation of tantalum oxide pores grown on tantalum by potentiodynamic anodic oxidation. J. Electroceramics.

[5] Wang G., Lee J.-H., Yang Y., Ruan G., Kim N.D., Ji Y., Tour J.M. (2015). Three-Dimensional Networked Nanoporous Ta_2_O_5_–x Memory System for Ultrahigh Density Storage. Nano Lett..

[6] Yu H., Zhu S., Yang X., Wang X., Sun H., Huo M. (2013). Synthesis of Coral-Like Tantalum Oxide Films via Anodization in Mixed Organic-Inorganic Electrolytes. PLoS ONE.

[7] Fialho L., Almeida Alves C.F., Marques L.S., Carvalho S. (2020). Development of stacked porous tantalum oxide layers by anodization. Appl. Surf. Sci..

[8] Wei W., Macak J.M., Shrestha N.K., Schmuki P. (2009). Thick Self-Ordered Nanoporous Ta_2_O_5_ Films with Long-Range Lateral Order. J. Electrochem. Soc..

[9] Barton J.E., Stender C.L., Li P., Odom T.W. (2009). Structural control of anodized tantalum oxide nanotubes. J. Mater. Chem..

[10] Anitha V.C., Goswami A., Sopha H., Nandan D., Gawande M.B., Cepe K., Ng S., Zboril R., Macak J.M. (2018). Pt nanoparticles decorated TiO_2_ nanotubes for the reduction of olefins. Appl. Mater. Today.

[11] Kim C., Kim S., Choi J., Lee J., Kang J.S., Sung Y.-E., Lee J., Choi W., Yoon J. (2014). Blue TiO_2_ Nanotube Array as an Oxidant Generating Novel Anode Material Fabricated by Simple Cathodic Polarization. Electrochim. Acta.

[12] Zheng Q., Lee H.-J., Lee J., Choi W., Park N.-B., Lee C. (2014). Electrochromic titania nanotube arrays for the enhanced photocatalytic degradation of phenol and pharmaceutical compounds. Chem. Eng. J..

[13] Nguyen N.T., Hwang I., Kondo T., Yanagishita T., Masuda H., Schmuki P. (2017). Optimizing TiO_2_ nanotube morphology for enhanced photocatalytic H_2_ evolution using single-walled and highly ordered TiO_2_ nanotubes decorated with dewetted Au nanoparticles. Electrochem. Commun..

[14] Altomare M., Cha G., Schmuki P. (2020). Anodic nanoporous niobium oxide layers grown in pure molten ortho-phosphoric acid. Electrochim. Acta.

[15] Agnieszka S., Tomasz G., Bożena Ł. (2019). Electrochemical Formation of Self-Organized Nanotubular Oxide Layers on Niobium (Review). Curr. Nanosci..

[16] Lu Q., Hashimoto T., Skeldon P., Thompson G.E., Habazaki H., Shimizu K. (2005). Nanoporous Anodic Niobium Oxide Formed in Phosphate/Glycerol Electrolyte. Electrochem. Solid-State Lett..

[17] Sieber I., Hildebrand H., Friedrich A., Schmuki P. (2005). Formation of self-organized niobium porous oxide on niobium. Electrochem. Commun..

[18] Martín-González M., Martinez-Moro R., Aguirre M.H., Flores E., Caballero-Calero O. (2020). Unravelling nanoporous anodic iron oxide formation. Electrochim. Acta.

[19] Liang F.-X., Liang L., Zhao X.-Y., Tong X.-W., Hu J.-G., Lin Y., Luo L.-B., Wu Y.-C. (2018). Mesoporous anodic α-Fe_2_O_3_ interferometer for organic vapor sensing application. RSC Adv..

[20] Prakasam H.E., Varghese O.K., Paulose M., Mor G.K., Grimes C.A. (2006). Synthesis and photoelectrochemical properties of nanoporous iron (III) oxide by potentiostatic anodization. Nanotechnology.

[21] Zhang B., Ni H., Chen R., Zhan W., Zhang C., Lei R., Zha Y. (2015). A two-step anodic method to fabricate self-organised nanopore arrays on stainless steel. Appl. Surf. Sci..

[22] Zhan W., Ni H., Chen R., Song X., Huo K., Fu J. (2012). Formation of nanopore arrays on stainless steel surface by anodization for visible-light photocatalytic degradation of organic pollutants. J. Mater. Res..

[23] Dhawan U., Pan H.-A., Shie M.-J., Chu Y.H., Huang G.S., Chen P.-C., Chen W.L. (2017). The Spatiotemporal Control of Osteoblast Cell Growth, Behavior, and Function Dictated by Nanostructured Stainless Steel Artificial Microenvironments. Nanoscale Res. Lett..

[24] Formentín P., Catalán Ú., Fernández-Castillejo S., Alba M., Baranowska M., Solà R., Pallarès J., Marsal L.F. (2015). Human aortic endothelial cell morphology influenced by topography of porous silicon substrates. J. Biomater. Appl..

[25] Rodriguez A., Molinero D., Valera E., Trifonov T., Marsal L.F., Pallarès J., Alcubilla R. (2005). Fabrication of silicon oxide microneedles from macroporous silicon. Sens. Actuators B Chem..

[26] Elia P., Nativ-Roth E., Zeiri Y., Porat Z. (2016). Determination of the average pore-size and total porosity in porous silicon layers by image processing of SEM micrographs. Microporous Mater..

[27] Xia X.H., Ashruf C.M.A., French P.J., Kelly J.J. (2000). Galvanic Cell Formation in Silicon/Metal Contacts:  The Effect on Silicon Surface Morphology. Chem. Mater..

[28] Noh K., Brammer K.S., Kim H., Jung S.-Y., Seong T.-Y., Jin S. (2011). Highly self-assembled nanotubular aluminum oxide by hard anodization. J. Mater. Res..

[29] Lee W., Park S.-J. (2014). Porous Anodic Aluminum Oxide: Anodization and Templated Synthesis of Functional Nanostructures. Chem. Rev..

[30] Balde M., Vena A., Sorli B. (2015). Fabrication of porous anodic aluminium oxide layers on paper for humidity sensors. Sens. Actuators B Chem..

[31] Zaraska L., Jaskuła M., Sulka G.D. (2016). Porous anodic alumina layers with modulated pore diameters formed by sequential anodizing in different electrolytes. Mater. Lett..

[32] Santos A., Balderrama V.S., Alba M., Formentín P., Ferré-Borrull J., Pallarès J., Marsal L.F. (2012). Nanoporous Anodic Alumina Barcodes: Toward Smart Optical Biosensors. Adv. Mater..

[33] Bertó-Roselló F., Xifré-Pérez E., Ferré-Borrull J., Marsal L.F. (2018). 3D-FDTD modelling of optical biosensing based on gold-coated nanoporous anodic alumina. Results Phys..

[34] Kumeria T., Rahman M.M., Santos A., Ferré-Borrull J., Marsal L.F., Losic D. (2014). Structural and Optical Nanoengineering of Nanoporous Anodic Alumina Rugate Filters for Real-Time and Label-Free Biosensing Applications. Anal. Chem..

[35] Santos A., Balderrama V.S., Alba M., Formentín P., Ferré-Borrull J., Pallarès J., Marsal L.F. (2012). Tunable Fabry-Pérot interferometer based on nanoporous anodic alumina for optical biosensing purposes. Nanoscale Res. Lett..

[36] Md Jani A.M., Losic D., Voelcker N.H. (2013). Nanoporous anodic aluminium oxide: Advances in surface engineering and emerging applications. Prog. Mater. Sci..

[37] Chen Z., Zhang J., Singh S., Peltier-Pain P., Thorson J.S., Hinds B.J. (2014). Functionalized Anodic Aluminum Oxide Membrane–Electrode System for Enzyme Immobilization. ACS Nano.

[38] Mey I., Steinem C., Janshoff A. (2012). Biomimetic functionalization of porous substrates: Towards model systems for cellular membranes. J. Mater. Chem..

[39] Aguilar-Sierra S.M., Ferré-Borrull J., Echeverría F.E., Marsal L.F. (2019). Titanium dioxide-coated nanoporous anodic alumina optical properties. Appl. Surf. Sci..

[40] Montero-Rama M.P., Viterisi A., Eckstein C., Ferré-Borrull J., Marsal L.F. (2019). In-situ removal of thick barrier layer in nanoporous anodic alumina by constant current Re-anodization. Surf. Coat. Technol..

[41] Law C.S., Lim S.Y., Liu L., Abell A.D., Marsal L.F., Santos A. (2020). Realization of high-quality optical nanoporous gradient-index filters by optimal combination of anodization conditions. Nanoscale.

[42] Matsumoto F., Harada M., Nishio K., Masuda H. (2005). Nanometer-Scale Patterning of DNA in Controlled Intervals on a Gold-Disk Array Fabricated Using Ideally Ordered Anodic Porous Alumina. Adv. Mater..

[43] Nishinaga O., Kikuchi T., Natsui S., Suzuki R.O. (2013). Rapid fabrication of self-ordered porous alumina with 10-/sub-10-nm-scale nanostructures by selenic acid anodizing. Sci. Rep..

[44] Kikuchi T., Nishinaga O., Natsui S., Suzuki R.O. (2014). Self-Ordering Behavior of Anodic Porous Alumina via Selenic Acid Anodizing. Electrochim. Acta.

[45] Schwirn K., Lee W., Hillebrand R., Steinhart M., Nielsch K., Gösele U. (2008). Self-Ordered Anodic Aluminum Oxide Formed by H2SO4 Hard Anodization. ACS Nano.

[46] Pashchanka M., Schneider J.J. (2016). Self-Ordering Regimes of Porous Anodic Alumina Layers Formed in Highly Diluted Sulfuric Acid Electrolytes. J. Phys. Chem. C.

[47] Nourmohammadi A., Asadabadi S.J., Yousefi M.H., Ghasemzadeh M. (2012). Photoluminescence emission of nanoporous anodic aluminum oxide films prepared in phosphoric acid. Nanoscale Res. Lett..

[48] Choudhari K.S., Kulkarni S.D., Santhosh C., George S.D. (2018). Photoluminescence enhancement and morphological properties of nanoporous anodic alumina prepared in oxalic acid with varying time and temperature. Microporous Mater..

[49] Leontiev A.P., Roslyakov I.V., Napolskii K.S. (2019). Complex influence of temperature on oxalic acid anodizing of aluminium. Electrochim. Acta.

[50] Stępniowski W.J., Forbot D., Norek M., Michalska-Domańska M., Król A. (2014). The impact of viscosity of the electrolyte on the formation of nanoporous anodic aluminum oxide. Electrochim. Acta.

[51] Santos A., Alba M., Rahman M.M., Formentín P., Ferré-Borrull J., Pallarès J., Marsal L.F. (2012). Structural tuning of photoluminescence in nanoporous anodic alumina by hard anodization in oxalic and malonic acids. Nanoscale Res. Lett..

[52] Lee W., Nielsch K., Gösele U. (2007). In Self-ordering behavior of nanoporous anodic aluminum oxide (AAO) in malonic acid anodization. Nanotechnology.

[53] Ma Y., Wen Y., Li J., Li Y., Zhang Z., Feng C., Sun R. (2016). Fabrication of Self-Ordered Alumina Films with Large Interpore Distance by Janus Anodization in Citric Acid. Sci. Rep..

[54] Takenaga A., Kikuchi T., Natsui S., Suzuki R.O. (2016). Exploration for the Self-ordering of Porous Alumina Fabricated via Anodizing in Etidronic Acid. Electrochim. Acta.

[55] Kikuchi T., Nishinaga O., Natsui S., Suzuki R.O. (2015). Fabrication of Self-Ordered Porous Alumina via Etidronic Acid Anodizing and Structural Color Generation from Submicrometer-Scale Dimple Array. Electrochim. Acta.

[56] Vrublevsky I.A., Chernyakova K.V., Ispas A., Bund A., Zavadski S. (2014). Optical properties of thin anodic alumina membranes formed in a solution of tartaric acid. Thin Solid Films.

[57] Law C.S., Lim S.Y., Abell A.D., Marsal L.F., Santos A. (2018). Structural tailoring of nanoporous anodic alumina optical microcavities for enhanced resonant recirculation of light. Nanoscale.

[58] Alvarez S.D., Li C.-P., Chiang C.E., Schuller I.K., Sailor M.J. (2009). A Label-Free Porous Alumina Interferometric Immunosensor. ACS Nano.

[59] Macias G., Hernández-Eguía L.P., Ferré-Borrull J., Pallares J., Marsal L.F. (2013). Gold-Coated Ordered Nanoporous Anodic Alumina Bilayers for Future Label-Free Interferometric Biosensors. ACS Appl. Mater. Interfaces.

[60] Eckstein C., Acosta L.K., Pol L., Xifré-Pérez E., Pallares J., Ferré-Borrull J., Marsal L.F. (2018). Nanoporous Anodic Alumina Surface Modification by Electrostatic, Covalent, and Immune Complexation Binding Investigated by Capillary Filling. ACS Appl. Mater. Interfaces.

[61] Rajeev G., Prieto Simon B., Marsal L.F., Voelcker N.H. (2018). Advances in Nanoporous Anodic Alumina-Based Biosensors to Detect Biomarkers of Clinical Significance: A Review. Adv. Healthc. Mater..

[62] Álvarez J., Sola L., Cretich M., Swann M.J., Gylfasson K.B., Volden T., Chiari M., Hill D. A real time immunoassay in alumina membranes. Proceedings of the SENSORS, 2014 IEEE.

[63] Álvarez J., Sola L., Cretich M., Swann M.J., Gylfason K.B., Volden T., Chiari M., Hill D. (2014). Real time optical immunosensing with flow-through porous alumina membranes. Sens. Actuators B Chem..

[64] Tanvir S., Pantigny J., Boulnois P., Pulvin S. (2009). Covalent immobilization of recombinant human cytochrome CYP2E1 and glucose-6-phosphate dehydrogenase in alumina membrane for drug screening applications. J. Membr. Sci..

[65] Jia R.-P., Shen Y., Luo H.-Q., Chen X.-G., Hu Z.-D., Xue D.-S. (2003). Photoluminescence spectra of human serum albumen and morin embedded in porous alumina membranes with ordered pore arrays. J. Phys. Condens. Matter..

[66] Qiu X., Xu X.-Y., Liang Y., Guo H. (2018). The molecularly imprinted polymer supported by anodic alumina oxide nanotubes membrane for efficient recognition of chloropropanols in vegetable oils. Food Chem..

[67] Carneiro J.O., Machado F., Pereira M., Teixeira V., Costa M.F., Ribeiro A., Cavaco-Paulo A., Samantilleke A.P. (2018). The influence of the morphological characteristics of nanoporous anodic aluminium oxide (AAO) structures on capacitive touch sensor performance: A biological application. RSC Adv..

[68] Hong C., Chu L.-A., Lai W., Chiang A.-S., Fang W. (2011). Implementation of a New Capacitive Touch Sensor Using the Nanoporous Anodic Aluminum Oxide (np-AAO) Structure. IEEE Sens. J..

[69] Yeh J.-H., Hong C., Hsu F.-M., Fang W. Novel temperature sensor implemented on nanoporous Anodic Aluminum Oxide template. Proceedings of the 2011 IEEE SENSORS.

[70] Salamat A., Islam T. (2020). Fabrication of an anodized porous alumina relative humidity sensor with improved sensitivity. Instrum. Sci. Technol..

[71] Lu Z., Ruan W., Yang J., Xu W., Zhao C., Zhao B. (2009). Deposition of Ag nanoparticles on porous anodic alumina for surface enhanced Raman scattering substrate. J. Raman Spectrosc..

[72] Sinn Aw M., Kurian M., Losic D. (2014). Non-eroding drug-releasing implants with ordered nanoporous and nanotubular structures: Concepts for controlling drug release. Biomater. Sci..

[73] Porta I.B.M., Xifré-Pérez E., Eckstein C., Ferré-Borrull J., Marsal L.F. (2017). 3D Nanoporous Anodic Alumina Structures for Sustained Drug Release. Nanomaterials.

[74] Saji V.S., Kumeria T., Gulati K., Prideaux M., Rahman S., Alsawat M., Santos A., Atkins G.J., Losic D. (2015). Localized drug delivery of selenium (Se) using nanoporous anodic aluminium oxide for bone implants. J. Mater. Chem. B.

[75] Porta-i-Batalla M., Eckstein C., Xifré-Pérez E., Formentín P., Ferré-Borrull J., Marsal L.F. (2016). Sustained, Controlled and Stimuli-Responsive Drug Release Systems Based on Nanoporous Anodic Alumina with Layer-by-Layer Polyelectrolyte. Nanoscale Res. Lett..

[76] Wang Y., Santos A., Kaur G., Evdokiou A., Losic D. (2014). Structurally engineered anodic alumina nanotubes as nano-carriers for delivery of anticancer therapeutics. Biomaterials.

[77] Li J., Liu Z., Huang G., An Z., Chen G., Zhang J., Li M., Liu R., Mei Y. (2014). Hierarchical nanoporous microtubes for high-speed catalytic microengines. NPG Asia Mater..

[78] Peng F., Tu Y., Wilson D.A. (2017). Micro/nanomotors towards in vivo application: Cell, tissue and biofluid. Chem. Soc. Rev..

[79] Mozalev A., Hubalek J. (2019). On-substrate porous-anodic-alumina-assisted gold nanostructure arrays: Meeting the challenges of various sizes and interfaces. Electrochim. Acta.

[80] Domagalski J.T., Xifre-Perez E., Santos A., Ferre-Borrull J., Marsal L.F. (2020). Tailor-engineered structural and physico-chemical properties of anodic alumina nanotubes by pulse anodization: A step forward. Microporous Mater..

[81] Ferré-Borrull J., Xifré-Pérez E., Pallarès J., Marsal L.F., Losic D., Santos A. (2015). Optical Properties of Nanoporous Anodic Alumina and Derived Applications. Nanoporous Alumina: Fabrication, Structure, Properties and Applications.

[82] Ito T., Matsuda Y., Jinba T., Asai N., Shimizu T., Shingubara S. (2017). Fabrication and characterization of nano porous lattice biosensor using anodic aluminum oxide substrate. Jpn. J. Appl. Phys..

[83] Law C.S., Marsal L.F., Santos A., Hussain C. (2020). 9—Electrochemically engineered nanoporous photonic crystal structures for optical sensing and biosensing. Handbook of Nanomaterials in Analytical Chemistry.

[84] Fu C., Gu Y., Wu Z., Wang Y., Xu S., Xu W. (2014). Surface-enhanced Raman scattering (SERS) biosensing based on nanoporous dielectric waveguide resonance. Sens. Actuators B Chem..

[85] Takmakov P., Vlassiouk I., Smirnov S. (2006). Hydrothermally shrunk alumina nanopores and their application to DNA sensing. Analyst.

[86] Hotta K., Yamaguchi A., Teramae N. (2012). Nanoporous Waveguide Sensor with Optimized Nanoarchitectures for Highly Sensitive Label-Free Biosensing. ACS Nano.

[87] Koutsioubas A.G., Spiliopoulos N., Anastassopoulos D., Vradis A.A., Priftis G.D. (2008). Nanoporous alumina enhanced surface plasmon resonance sensors. J. Appl. Phys..

[88] Dhathathreyan A. (2011). Real-Time Monitoring of Invertase Activity Immobilized in Nanoporous Aluminum Oxide. J. Phys. Chem. B.

[89] Chen R., Du X., Cui Y., Zhang X., Ge Q., Dong J., Zhao X. (2020). Vertical Flow Assay for Inflammatory Biomarkers Based on Nanofluidic Channel Array and SERS Nanotags. Small.

[90] Lazzara T.D., Mey I., Steinem C., Janshoff A. (2011). Benefits and Limitations of Porous Substrates as Biosensors for Protein Adsorption. Anal. Chem..

[91] Fan Y., Hotta K., Yamaguchi A., Ding Y., He Y., Teramae N., Sun S., Ma H. (2014). Highly sensitive real-time detection of DNA hybridization by using nanoporous waveguide fluorescence spectroscopy. Appl. Phys. Lett..

[92] Yamaguchi A., Hotta K., Teramae N. (2009). Optical Waveguide Sensor Based on a Porous Anodic Alumina/Aluminum Multilayer Film. Anal. Chem..

[93] Hotta K., Yamaguchi A., Teramae N. (2012). Deposition of Polyelectrolyte Multilayer Film on a Nanoporous Alumina Membrane for Stable Label-Free Optical Biosensing. J. Phys. Chem. C.

[94] Fan Y., Hotta K., Yamaguchi A., Teramae N. (2012). Enhanced fluorescence in a nanoporous waveguide and its quantitative analysis. Opt. Express.

[95] Ji N., Ruan W., Wang C., Lu Z., Zhao B. (2009). Fabrication of Silver Decorated Anodic Aluminum Oxide Substrate and Its Optical Properties on Surface-Enhanced Raman Scattering and Thin Film Interference. Langmuir.

[96] Hiep H.M., Yoshikawa H., Tamiya E. (2010). Interference Localized Surface Plasmon Resonance Nanosensor Tailored for the Detection of Specific Biomolecular Interactions. Anal. Chem..

[97] Kim S.-W., Lee J.-S., Lee S.-W., Kang B.-H., Kwon J.-B., Kim O.-S., Kim J.-S., Kim E.-S., Kwon D.-H., Kang S.-W. (2017). Easy-to-Fabricate and High-Sensitivity LSPR Type Specific Protein Detection Sensor Using AAO Nano-Pore Size Control. Sensors.

[98] Lee J.-S., Kim S.-W., Jang E.-Y., Kang B.-H., Lee S.-W., Sai-Anand G., Lee S.-H., Kwon D.-H., Kang S.-W. (2015). Rapid and Sensitive Detection of Lung Cancer Biomarker Using Nanoporous Biosensor Based on Localized Surface Plasmon Resonance Coupled with Interferometry. J. Nanomater..

[99] Yeom S.H., Kim O.G., Kang B.H., Kim K.J., Yuan H., Kwon D.H., Kim H.R., Kang S.W. (2011). Highly sensitive nano-porous lattice biosensor based on localized surface plasmon resonance and interference. Opt. Express.

[100] Kim D.-K., Kerman K., Hiep H.M., Saito M., Yamamura S., Takamura Y., Kwon Y.-S., Tamiya E. (2008). Label-free optical detection of aptamer–protein interactions using gold-capped oxide nanostructures. Anal. Biochem..

[101] Kim D.-K., Kerman K., Saito M., Sathuluri R.R., Endo T., Yamamura S., Kwon Y.-S., Tamiya E. (2007). Label-Free DNA Biosensor Based on Localized Surface Plasmon Resonance Coupled with Interferometry. Anal. Chem..

[102] Pla L., Xifré-Pérez E., Ribes À., Aznar E., Marcos M.D., Marsal L.F., Martínez-Máñez R., Sancenón F. (2017). A Mycoplasma Genomic DNA Probe using Gated Nanoporous Anodic Alumina. ChemPlusChem.

[103] Yu Z., Lei Y., Yu W., Cheng J., Xing J., Zheng X., Zhan Z., Wang B., Guo C. (2019). Fluorescence enhanced lab-on-a-chip patterned using a hybrid technique of femtosecond laser direct writing and anodized aluminum oxide porous nanostructuring. Nanoscale Adv..

[104] Takmakov P., Vlassiouk I., Smirnov S. (2006). Application of anodized aluminum in fluorescence detection of biological species. Anal. BioAnal. Chem..

[105] Santos A., Macías G., Ferré-Borrull J., Pallarès J., Marsal L.F. (2012). Photoluminescent Enzymatic Sensor Based on Nanoporous Anodic Alumina. ACS Appl. Mater. Interfaces.

[106] Jia R.P., Shen Y., Luo H.Q., Chen X.G., Hu Z.D., Xue D.S. (2004). Enhanced photoluminescence properties of morin and trypsin absorbed on porous alumina films with ordered pores array. Solid State Commun..

[107] Li Y.B., Zheng M.J., Ma L. (2007). High-speed growth and photoluminescence of porous anodic alumina films with controllable interpore distances over a large range. Appl. Phys. Lett..

[108] Zhang W., Tian Q., Chen Z., Zhao C., Chai H., Wu Q., Li W., Chen X., Deng Y., Song Y. (2020). Arrayed nanopore silver thin films for surface-enhanced Raman scattering. RSC Adv..

[109] Toccafondi C., Thorat S., La Rocca R., Scarpellini A., Salerno M., Dante S., Das G. (2014). Multifunctional substrates of thin porous alumina for cell biosensors. J. Mater. Sci.: Mater. Med..

[110] Velleman L., Bruneel J.-L., Guillaume F., Losic D., Shapter J.G. (2011). Raman spectroscopy probing of self-assembled monolayers inside the pores of gold nanotube membranes. Phys. Chem. Chem. Phys..

[111] Kodiyath R., Malak S.T., Combs Z.A., Koenig T., Mahmoud M.A., El-Sayed M.A., Tsukruk V.V. (2013). Assemblies of silver nanocubes for highly sensitive SERS chemical vapor detection. J. Mater. Chem. A.

[112] Ko H., Tsukruk V.V. (2008). Nanoparticle-Decorated Nanocanals for Surface-Enhanced Raman Scattering. Small.

[113] Lee S.J., Guan Z., Xu H., Moskovits M. (2007). Surface-Enhanced Raman Spectroscopy and Nanogeometry:  The Plasmonic Origin of SERS. J. Phys. Chem. C.

[114] Hao Q., Huang H., Fan X., Hou X., Yin Y., Li W., Si L., Nan H., Wang H., Mei Y. (2017). Facile design of ultra-thin anodic aluminum oxide membranes for the fabrication of plasmonic nanoarrays. Nanotechnology.

[115] Kumeria T., Losic D. (2011). Reflective interferometric gas sensing using nanoporous anodic aluminium oxide (AAO). Phys. Status Solidi-R.

[116] Pan S., Rothberg L.J. (2003). Interferometric Sensing of Biomolecular Binding Using Nanoporous Aluminum Oxide Templates. Nano Lett..

[117] Dronov R., Jane A., Shapter J.G., Hodges A., Voelcker N.H. (2011). Nanoporous alumina-based interferometric transducers ennobled. Nanoscale.

[118] Mutalib Md Jani A., Anglin E.J., McInnes S.J.P., Losic D., Shapter J.G., Voelcker N.H. (2009). Nanoporous anodic aluminium oxide membranes with layered surface chemistry. ChemComm.

[119] Kumeria T., Losic D. (2012). Controlling interferometric properties of nanoporous anodic aluminium oxide. Nanoscale Res. Lett..

[120] Shi W., Shen Y., Ge D., Xue M., Cao H., Huang S., Wang J., Zhang G., Zhang F. (2008). Functionalized anodic aluminum oxide (AAO) membranes for affinity protein separation. J. Membr. Sci..

[121] Rajeev G., Xifre-Perez E., Prieto Simon B., Cowin A.J., Marsal L.F., Voelcker N.H. (2018). A label-free optical biosensor based on nanoporous anodic alumina for tumour necrosis factor-alpha detection in chronic wounds. Sens. Actuators B Chem..

[122] Amouzadeh Tabrizi M., Ferré-Borrull J., Marsal L.F. (2019). Highly sensitive aptasensor based on interferometric reflectance spectroscopy for the determination of amyloid β as an Alzheimer’s disease biomarkers using nanoporous anodic alumina. Biosens. Bioelectron..

[123] Pol L., Acosta L.K., Ferré-Borrull J., Marsal L.F. (2019). Aptamer-Based Nanoporous Anodic Alumina Interferometric Biosensor for Real-Time Thrombin Detection. Sensors.

[124] Nemati M., Santos A., Kumeria T., Losic D. (2015). Label-Free Real-Time Quantification of Enzyme Levels by Interferometric Spectroscopy Combined with Gelatin-Modified Nanoporous Anodic Alumina Photonic Films. Anal. Chem..

[125] Lau K.H.A., Duran H., Knoll W. (2009). In situ Characterization of N-Carboxy Anhydride Polymerization in Nanoporous Anodic Alumina. J. Phys. Chem. B.

[126] Metkar S.K., Girigoswami K. (2019). Diagnostic biosensors in medicine—A review. Biocatal. Agric. Biotechnol..

[127] Leva-Bueno J., Peyman S.A., Millner P.A. (2020). A review on impedimetric immunosensors for pathogen and biomarker detection. Med. Microbiol. Immunol..

[128] Kumeria T., Kurkuri M.D., Diener K.R., Parkinson L., Losic D. (2012). Label-free reflectometric interference microchip biosensor based on nanoporous alumina for detection of circulating tumour cells. Biosens. Bioelectron..

[129] Yeom S.-H., Han M.-E., Kang B.-H., Kim K.-J., Yuan H., Eum N.-S., Kang S.-W. (2013). Enhancement of the sensitivity of LSPR-based CRP immunosensors by Au nanoparticle antibody conjugation. Sens. Actuators B Chem..

[130] Kaur H., Shorie M. (2019). Nanomaterial based aptasensors for clinical and environmental diagnostic applications. Nanoscale Adv..

[131] Thiviyanathan V., Gorenstein D.G. (2012). Aptamers and the next generation of diagnostic reagents. Proteom. Clin. Appl..

[132] Ribes À., Aznar E., Bernardos A., Marcos M.D., Amorós P., Martínez-Máñez R., Sancenón F. (2017). Fluorogenic Sensing of Carcinogenic Bisphenol A using Aptamer-Capped Mesoporous Silica Nanoparticles. Chemistry.

[133] Liang G., Man Y., Li A., Jin X., Liu X., Pan L. (2017). DNAzyme-based biosensor for detection of lead ion: A review. Microchem. J..

[134] Amouzadeh Tabrizi M., Ferré-Borrull J., Marsal L.F. (2020). Highly sensitive remote biosensor for the determination of lead (II) ions by using nanoporous anodic alumina modified with DNAzyme. Sens. Actuators B Chem..

[135] Zhu X., Gao X., Liu Q., Lin Z., Qiu B., Chen G. (2011). Pb2+-introduced activation of horseradish peroxidase (HRP)-mimicking DNAzyme. ChemComm.

[136] Kumeria T., Rahman M.M., Santos A., Ferré-Borrull J., Marsal L.F., Losic D. (2014). Nanoporous Anodic Alumina Rugate Filters for Sensing of Ionic Mercury: Toward Environmental Point-of-Analysis Systems. ACS Appl. Mater. Interfaces.

[137] Kaur S., Law C.S., Williamson N.H., Kempson I., Popat A., Kumeria T., Santos A. (2019). Environmental Copper Sensor Based on Polyethylenimine-Functionalized Nanoporous Anodic Alumina Interferometers. Anal. Chem..

[138] Kumeria T., Santos A., Losic D. (2013). Ultrasensitive Nanoporous Interferometric Sensor for Label-Free Detection of Gold(III) Ions. ACS Appl. Mater. Interfaces.

[139] Law C.S., Lim S.Y., Abell A.D., Santos A. (2018). Real-Time Binding Monitoring between Human Blood Proteins and Heavy Metal Ions in Nanoporous Anodic Alumina Photonic Crystals. Anal. Chem..

[140] Eckstein C., Law C.S., Lim S.Y., Kaur S., Kumeria T., Ferré-Borrull J., Abell A.D., Marsal L.F., Santos A. (2019). Nanoporous photonic crystals with tailored surface chemistry for ionic copper sensing. J. Mater. Chem. C.

[141] Chen Y., Santos A., Wang Y., Kumeria T., Wang C., Li J., Losic D. (2015). Interferometric nanoporous anodic alumina photonic coatings for optical sensing. Nanoscale.

[142] Amouzadeh Tabrizi M., Shamsipur M. (2015). A label-free electrochemical DNA biosensor based on covalent immobilization of salmonella DNA sequences on the nanoporous glassy carbon electrode. Biosens. Bioelectron..

[143] Gong Q., Han H., Yang H., Zhang M., Sun X., Liang Y., Liu Z., Zhang W., Qiao J. (2019). Sensitive electrochemical DNA sensor for the detection of HIV based on a polyaniline/graphene nanocomposite. J. Mater..

[144] Ribes À., Aznar E., Santiago-Felipe S., Xifre-Perez E., Tormo-Mas M.Á., Pemán J., Marsal L.F., Martínez-Máñez R. (2019). Selective and Sensitive Probe Based in Oligonucleotide-Capped Nanoporous Alumina for the Rapid Screening of Infection Produced by Candida albicans. ACS Sensors.

[145] Amouzadeh Tabrizi M., Ferré-Borrull J., Marsal L.F. (2020). Remote sensing of Salmonella-specific DNA fragment by using nanoporous alumina modified with the single-strand DNA probe. Sens. Actuators B Chem..

[146] Rohs R., Sklenar H., Lavery R., Röder B. (2000). Methylene Blue Binding to DNA with Alternating GC Base Sequence:  A Modeling Study. J. Am. Chem. Soc..

[147] Lin X., Ni Y., Kokot S. (2015). An electrochemical DNA-sensor developed with the use of methylene blue as a redox indicator for the detection of DNA damage induced by endocrine-disrupting compounds. Anal. Chim. Acta.

[148] Liu Q., Wang J., Boyd B.J. (2015). Peptide-based biosensors. Talanta.

[149] Amouzadeh Tabrizi M., Ferré-Borrull J., Marsal L.F. (2020). An optical biosensor for the determination of cathepsin B as a cancer-associated enzyme using nanoporous anodic alumina modified with human serum albumin-thionine. Mikrochim. Acta.

[150] Nguyen H.H., Lee S.H., Lee U.J., Fermin C.D., Kim M. (2019). Immobilized Enzymes in Biosensor Applications. Materials.

[151] Amouzadeh Tabrizi M., Ferré-Borrull J., Marsal L.F. (2020). Highly sensitive IRS based biosensor for the determination of cytochrome c as a cancer marker by using nanoporous anodic alumina modified with trypsin. Biosens. Bioelectron..

[152] Zhang L., Qin H., Cui W., Zhou Y., Du J. (2016). Label–free, turn–on fluorescent sensor for trypsin activity assay and inhibitor screening. Talanta.

[153] Chen J., Zhang Y., Cheng M., Guo Y., Šponer J., Monchaud D., Mergny J.-L., Ju H., Zhou J. (2018). How Proximal Nucleobases Regulate the Catalytic Activity of G-Quadruplex/Hemin DNAzymes. ACS Catal..

[154] Amouzadeh Tabrizi M., Ferré-Borrull J., Marsal L.F. (2020). Nanoporous Anodic Alumina As a Three-dimensional Nanostructured material for the Remote Optical Sensing of Urea. ECS Meet. Abstr..

[155] Mazzei L., Cianci M., Gonzalez Vara A., Ciurli S. (2018). The structure of urease inactivated by Ag(i): A new paradigm for enzyme inhibition by heavy metals. Dalton Trans..

[156] Amouzadeh Tabrizi M., Ferré-Borrull J., Marsal L.F. (2020). Remote biosensor for the determination of trypsin by using nanoporous anodic alumina as a three-dimensional nanostructured material. Sci. Rep..

[157] Chen Y., Santos A., Wang Y., Kumeria T., Li J., Wang C., Losic D. (2015). Biomimetic Nanoporous Anodic Alumina Distributed Bragg Reflectors in the Form of Films and Microsized Particles for Sensing Applications. ACS Appl. Mater. Interfaces.

[158] Macias G., Ferré-Borrull J., Pallarès J., Marsal L.F. (2015). Effect of pore diameter in nanoporous anodic alumina optical biosensors. Analyst.

[159] Pol L., Eckstein C., Acosta L.K., Xifré-Pérez E., Ferré-Borrull J., Marsal L.F. (2019). Real-Time Monitoring of Biotinylated Molecules Detection Dynamics in Nanoporous Anodic Alumina for Bio-Sensing. Nanomaterials.

[160] Acosta L.K., Bertó-Roselló F., Xifre-Perez E., Santos A., Ferré-Borrull J., Marsal L.F. (2019). Stacked Nanoporous Anodic Alumina Gradient-Index Filters with Tunable Multispectral Photonic Stopbands as Sensing Platforms. ACS Appl. Mater. Interfaces.

[161] Acosta L.K., Bertó-Roselló F., Xifre-Perez E., Law C.S., Santos A., Ferré-Borrull J., Marsal L.F. (2020). Tunable Nanoporous Anodic Alumina Photonic Crystals by Gaussian Pulse Anodization. ACS Appl. Mater. Interfaces.

[162] Santos A., Kumeria T., Losic D. (2013). Optically Optimized Photoluminescent and Interferometric Biosensors Based on Nanoporous Anodic Alumina: A Comparison. Anal. Chem..

[163] Ferro L.M.M., Lemos S.G., Ferreira M., Trivinho-Strixino F. (2017). Use of multivariate analysis on Fabry-Pérot interference spectra of nanoporous anodic alumina (NAA) for optical sensors purposes. Sens. Actuators B Chem..

[164] Nemati M., Santos A., Losic D. (2018). Fabrication and Optimization of Bilayered Nanoporous Anodic Alumina Structures as Multi-Point Interferometric Sensing Platform. Sensors.

[165] Dai J., Baker G.L., Bruening M.L. (2006). Use of Porous Membranes Modified with Polyelectrolyte Multilayers as Substrates for Protein Arrays with Low Nonspecific Adsorption. Anal. Chem..

[166] Ribes À., Xifré -Pérez E., Aznar E., Sancenón F., Pardo T., Marsal L.F., Martínez-Máñez R. (2016). Molecular gated nanoporous anodic alumina for the detection of cocaine. Sci. Rep..

[167] Pla L., Santiago-Felipe S., Tormo-Mas M.Á., Pemán J., Sancenón F., Aznar E., Martínez-Máñez R. (2020). Aptamer-Capped nanoporous anodic alumina for Staphylococcus aureus detection. Sens. Actuators B Chem..

